# Rest, Repair, Repeat: The Complex Relationship of Autophagy and Sleep

**DOI:** 10.1016/j.jmb.2025.169227

**Published:** 2025-05-21

**Authors:** Halvor Ullern, Paulina Schnur, Charlotte N Boccara, Helene Knævelsrud

**Affiliations:** 1 Department of Molecular Medicine, Institute of Basic Medical Sciences, University of Oslo, Oslo, Norway; 2 Centre for Cancer Cell Reprogramming, Institute of Clinical Medicine, Faculty of Medicine, University of Oslo, Norway; 3 Norwegian Centre for Molecular Biosciences and Medicine (NCMBM), University of Oslo, Oslo, Norway; 4 Department of Neurology, Clinical Neuroscience, Oslo University Hospital (OUS), Norway; 5 Department of Molecular Cell Biology, Institute for Cancer Research, Oslo University Hospital, Norway

**Keywords:** Autophagy, sleep, homeostasis, circadian rhythm, macroautophagy, neurosciences

## Abstract

Autophagy and sleep are two evolutionary conserved mechanisms across the animal kingdom. Autophagy is a pathway for the degradation of cytoplasmic material in the lysosome, playing important roles in the homeostasis and health of the organism. On the other hand, sleep is a homeostatically regulated state with numerous presumed essential roles, including the restoration of tissue and physiological functions, such as brain waste clearance via the activation of the glymphatic systems. Given that sleep and autophagy are crucial processes tightly linked to homeostasis and maintenance of good health, understanding how they interact is of great interest, especially as sleep quality decreases in our modern 24-hour societies. Autophagy represents a promising target for therapeutic interventions in this context. Here, we review the contrasted and complementary roles of autophagy and sleep in maintaining homeostasis. Specifically, we focus on recent evidence suggesting that sleep impairment may increase autophagy, while autophagosome levels may modulate the amount of sleep. We discuss outstanding questions at the intersection of these two fields, highlighting methodological shortcomings in the current literature. Overcoming these limitations will be instrumental to design new experiments with the aim of answering one of the greatest mysteries of our time – why do we sleep?

## Graphical abstract

**Figure:**
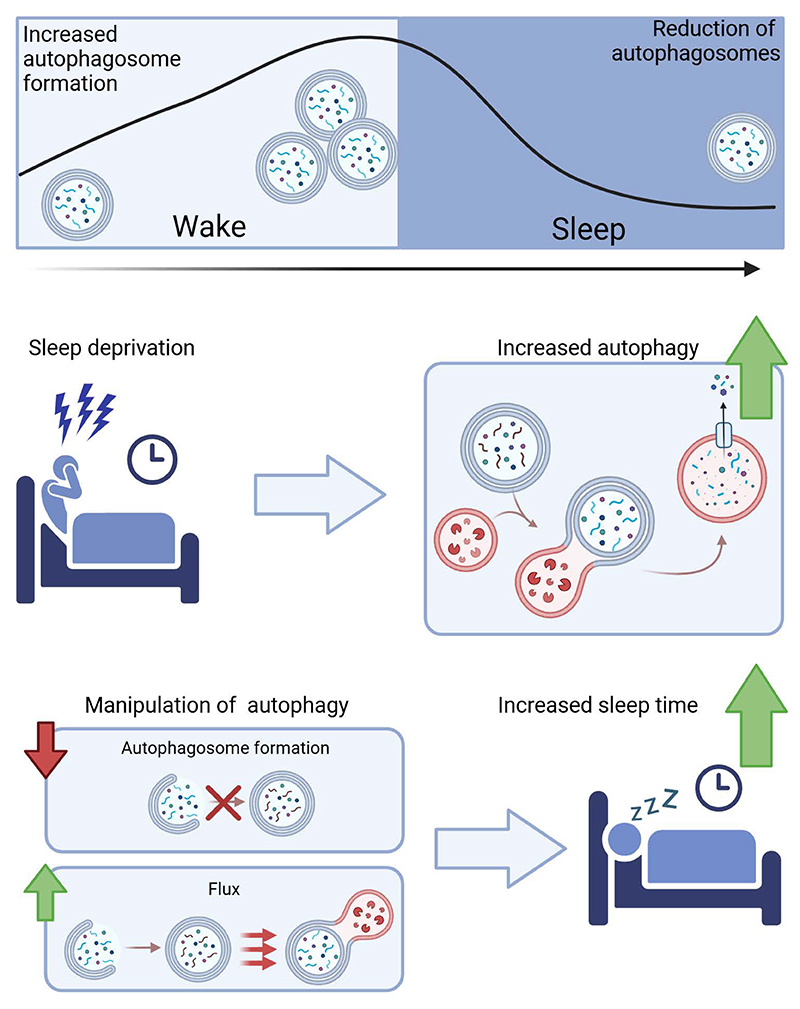

## Abbreviations

ATG: Autophagy-related
ATP: Adenosine triphosphate
AMPK: Adenosine monophosphate activated protein kinase
AQP4: Channel aquaporin-4
aus: argus
BafA1: BafilomycinA1
bchs: blue cheese 58
BMAL1: Basic helix-loop-helix ARNT-like protein 1
CLOCK: Circadian locomotor output cycles kaput
CMA: Chaperone-mediated autophagy
CRH: corticotropin-releasing hormone
CRFR: Corticotropin-releasing hormone receptor
CRISPR: clustered regularly interspaced short palindromic repeats
Cry: Cryptochrome
CSF: cerebrospinal fluid
EEG: electroencephalogram
EM: electron microscopy
EOG: electro-oculography
GABA: Gamma-aminobutyric acid
GABARAP: GABA type A receptor associated protein
GABARAPL1: GABARAP-like1
GABARAPL2: GABARAP-like2
GFP: Green fluorescent protein
GH: Growth hormone
HPA: hypothalamic-pituitary-adrenal
IF: Immunofluorescence
IHC: Immunohistochemistry
ipRGCs: intrinsically photoreceptive retinal ganglion cells
ISF: interstitial fluid
LAMP1: Lysosomal-associated membrane protein 1
LC3: Microtubule-associated protein 1A/1B-light chain 3
lLNv: large ventrolateral neurons
MMPM: Modified multiple platform method
mTORC1: mammalian target of rapamycin complex 1
NAD: Nicotinamide adenine dinucleotide
NMDA: N-methyl-D-aspartate
NREM: Non rapid eye movement
PDF: pigment dispersing factor
REM: Rapid eye movement
ROS: reactive oxygen species
SCN: Suprachiasmatic nucleus
SIRT1: Sirtuin 1
SR: Sleep restriction
sLNv: small ventrolateral neurons
qPCR: Quantitative polymerase chain reaction
TEM: Transmission electron microscopy
TFEB: transcription factor EB
TMS: transcranial magnetic stimulation
ULK1: Unc-51-like kinase 1
WB: Western Blot

## Relevance of potential interaction between autophagy and sleep

Understanding the functions of sleep is one of the greatest scientific challenges of our time. As reports accumulate, the scientific community is progressively realizing the extent to which sleep is a pillar of our health [1]. It serves numerous essential functions, including tissue and homeostatic function restoration, cognitive and maturation processes, as well as metabolic, immune, and hormonal regulation [2–4]. Yet, despite decades of research, we still know very little about the mechanisms by which sleep quality impacts our health.

Autophagy is a crucial cellular mechanism and important in health and disease [5]. In popular terms, autophagy is thought of as a process during which cells “clean out their garbage”. It is important for homeostatic processes and is regulated by the circadian rhythm [6] [7]. As such, autophagy and sleep are very well positioned to functionally interact.

Having a better grasp on the precise role sleep plays in maintaining brain and bodily health has never been more critical to address what the World Health Organization describes as an emerging global epidemic with both health, social and economic consequences [8, 9]. Crucial new studies point to increased sleep dysfunctions in patients diagnosed with cancer, psychiatric or metabolic disorders [10–12]. Sleep disturbances may actually be predictive of the severity of some diagnosis (e.g., Alzheimer disease, autism spectrum disorders or fibromyalgia) [13–15]. Deciphering the exact relationship between sleep and autophagy, and how the interplay affects health and disease, could provide new inroads for therapies, and improve overall quality of life.

Here we provide the necessary background for sleep researchers and autophagy researchers to further address these questions. We first present autophagy with a simplified overview of the core molecular machinery, a summary of the main methods used to assess autophagy, including their limitations, and an outline of the circadian regulation of autophagy. Second, we outline the characteristics and regulation of sleep, the main models for sleep manipulation, as well as the metabolic changes and waste clearance during sleep. With this background we summarize our current understanding of autophagy in physiological sleep, the role of autophagy upon sleep impairment and the impact of autophagy modulation on sleep. Finally, we highlight methodological limitations and outstanding questions at the intersection of these two fields.

## Autophagy – definitions and functions

Autophagy, meaning self-eating, consists of a set of conserved pathways for degrading cytoplasmatic material in the lysosome: macroautophagy, microautophagy and chaperone-mediated autophagy (CMA) [16]. Macroautophagy is characterized by the presence of the autophagosome – a double membrane vesicle that can sequester large parts of the cytoplasm, including whole organelles - and is the best understood of these pathways so far. Since only macroautophagy has been investigated in the context of sleep, we will refer to macroautophagy simply as “autophagy” in this review.

Autophagy is essential for recycling building blocks and providing energy under conditions when nutrients are limited, such as during starvation or at certain stages of development. Another crucial function fulfilled by autophagy is the selective degrading of potentially harmful cellular components, including misfolded or damaged proteins, whole dysfunctional organelles or even foreign substances like viruses and bacteria [17]. Autophagy operates continuously at a basal level in order to maintain cellular homeostasis [18], but it can be up- and down-regulated by nutritional, metabolic, hormonal, physical or chemical stimuli [19]. In general, autophagy is thought to promote a healthy lifespan [5, 20]. Dysregulated autophagy is linked to several pathologies, including neurodegenerative disorders and cancer, as well as inflammatory and autoimmune diseases [5].

## The autophagy machinery

**Figure 1:**
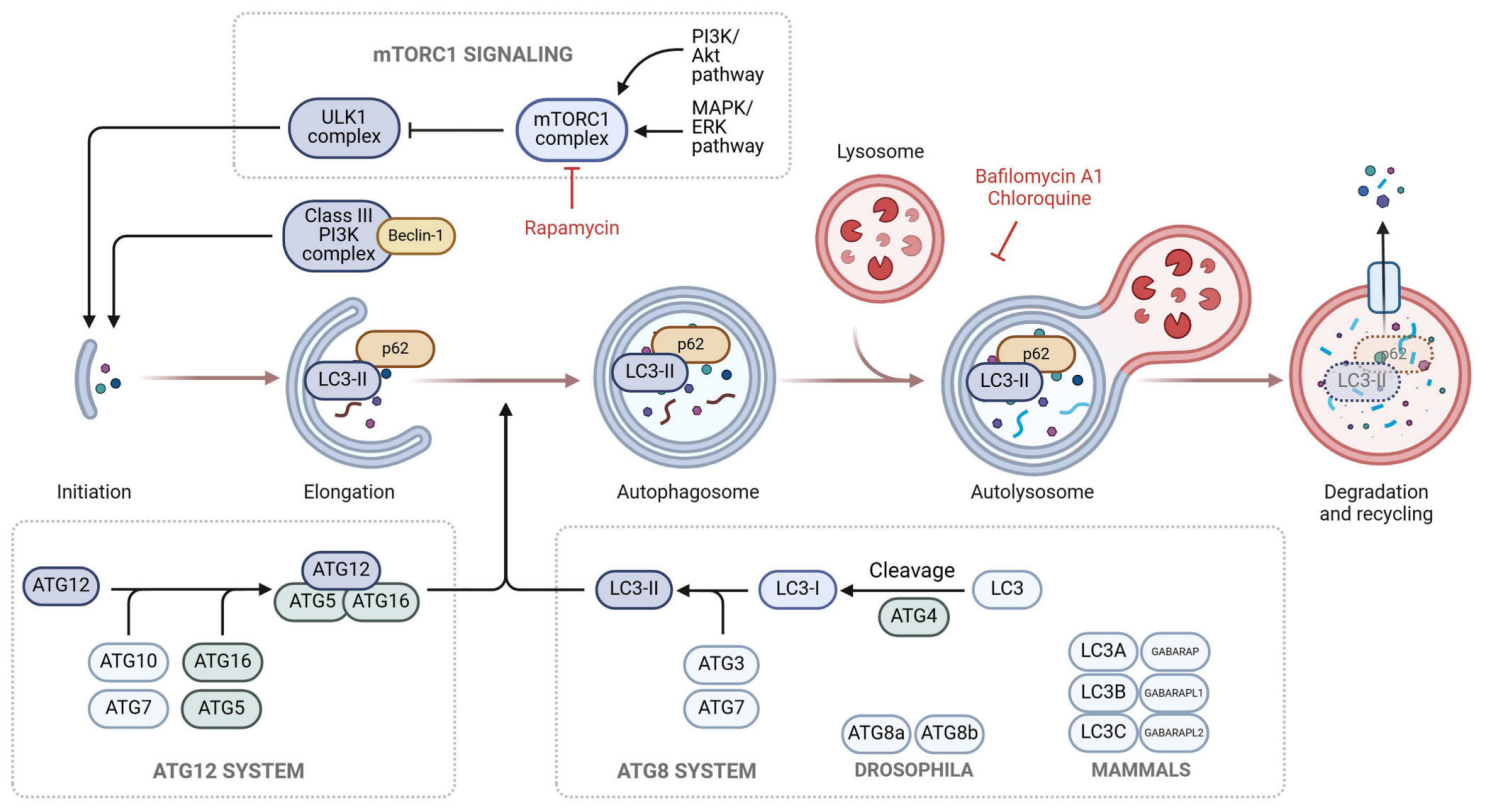
Autophagy overview. A simplified overview of some of the main proteins and protein complexes involved in the regulation of autophagosome formation. Note that lipidated ATG8-family proteins (such as LC3, denoted as LC3-II when lipidated) and the autophagy cargo receptor p62 will themselves be degraded in the autolysosomes.

Autophagy is initiated by the formation of a phagophore which elongates and eventually closes to form the double membrane vesicle called an autophagosome (Fig. 1). The autophagosome will mature and fuse with a lysosome to create an autolysosome where the cargo can be degraded [17]. mTORC1 is an important regulator of autophagy, primarily through inhibitory phosphorylation of the ULK1/Atg1 complex. Inhibition of mTORC1, for instance through amino acid starvation or by the drug rapamycin, allows for the activation of the ULK1/Atg1 complex as well as the translocation of the transcription factor EB (TFEB) and E3 (TFE3) to the nucleus, where they can activate genes related to autophagy and lysosomal biogenesis [21–23]. In addition, there are several ways to induce autophagy independently of ULK1/Atg1, such as through direct recruitment of FIP200 [24], through ADUK/ULK3 [25], or through increased VPS34 activity [26].

The autophagy process is controlled by a set of evolutionarily conserved genes, termed Autophagy-related *(ATG)* genes [27]. The main regulator of autophagy initiation is the ULK1/Atg1 kinase complex, which phosphorylates many of the other ATG proteins. Nucleation of the autophagosome requires the Beclin1/PI3KC3 complex PI3KC3-C1, which includes ATG14. Two important ubiquitin-like conjugation systems are involved in the autophagosome membrane elongation and closure, the ATG12 and the ATG8 system (Fig. 1). The ATG12-ATG5-ATG16L1 complex assists in conjugation of ATG8 proteins to phosphatidylethanolamine [28, 29]. In this reaction, cytosolic ATG8 family proteins such as LC3 are lipidated and attached to the membrane of the forming autophagosomes. Six different ATG8 family proteins exist in mammals: LC3A/MAP1LC3A (microtubule-associated protein 1 light chain 3 alpha), LC3B (MAP1LC3 beta), LC3C (MAP1LC3 gamma), GABARAP (GABA type A receptor associated protein), GABARAPL1 (GABARAP-like1) and GABARAPL2 (GABARAP-like2) [30], whereof LC3B (often referred to just as LC3) has so far been most commonly used to assess autophagy (Fig. 1). Although the core autophagy machinery encoded by the *ATG* genes are important for autophagy, just like there are ways to induce autophagy independently of ULK1/Atg1, alternative autophagy has been described to take place even in the absence of specific components of the core machinery [31]. On the other hand, there are numerous examples of non-canonical roles of autophagy proteins [32]. Since ATG8 proteins are often used as markers of autophagy, it is essential to know that ATG8 lipidation also serves several autophagy-independent functions [33, 34].

Autophagy substrates or cargo can be recognized by an autophagy receptor and thereby be targeted for degradation in the lysosome [35]. p62/SQSTM1 is a well-characterized cargo receptor for selective degradation of ubiquitinated misfolded proteins by autophagy [36]. It binds to ATG8-proteins through a LIR-motif [37] and is therefore itself turned over by autophagy (Fig. 1). Additionally, p62 can recruit the ULK1 kinase complex through a direct interaction with the subunit FIP200 and create fluid-like ubiquitinated-positive condensates [38]. Furthermore, some autophagy receptor proteins, like p62, can bind the Atg12-Atg5-Atg16 complex to increase Atg8-conjugation [39].

## Methods for monitoring autophagy

There are many different methods used to measure autophagy, which each have their own strengths and weaknesses that are detailed in the community guidelines [40]. The general recommendation is that more than one method should be used to assess autophagy. Some of the main methods that have been employed in the context of sleep are summarized in Table 1.

Note that to properly measure LC3 flux by IHC, IF or immunoblotting, it is imperative to include lysosomal blockade. This is performed using pharmacological agents like BafilomycinA1 (BafA1) or Chloroquine, the latter being better suited in vivo [40]. Such lysosomal blockade prevents the degradation of LC3-II, so that comparing LC3-II with and without the inhibitor, allows the quantification of LC3-II generated over the time course of the experiment. The interpretation of these experiments requires careful consideration since increased LC3-II levels can indicate either an increase in autophagy induction or an impairment of the autophagy flux. The kinetics of LC3 lipidation and turnover also varies between different cell types and tissues. In addition, LC3 flux might not reflect actual sequestration and degradation of cargo [41], partly because of the non-canonical LC3 functions mentioned above [33, 34]. To address these issues, different *ATG* knockdown or knockout models should be used and other autophagy markers such as p62 should be included in some assays. Inhibition of autophagy correlates with increased p62 levels, and vice versa for activation of autophagy [42]. As for LC3, changes in p62 levels are cell and tissue dependent. Furthermore, p62 has several autophagy-independent functions. Other markers that can complete the picture of whether and how autophagy is activated or inactivated would include ULK1, Beclin1, AMPK, mTOR (and some of its targets such as S6K, 4EBP, TFEB), Atg9, Atg12-Atg5, Atg14 and Atg16L1 [40].

## Circadian rhythms of autophagy

Circadian rhythms are the cyclic behavioral, physiological, and molecular changes an organism naturally experiences over a 24-hours cycle. They roughly follow the light/dark cycle resulting from earth rotation. Intrinsic circadian clocks can be observed in most cells from unicellular eukaryotes to multicellular organisms [43]. These intrinsic clocks pace molecular and cellular cyclic processes through the expression of clock genes. In many animals, these peripherical clocks are synchronized by a master clock located in a dedicated set of neurons that coordinates biological processes with external environmental cues. There is increasing evidence that impaired ability to maintain circadian rhythms negatively affects health and can promote disease [44]. There is extensive crosstalk of circadian clocks with metabolic and hormonal processes [45], leading to regular daily variation of gene expression across tissues such as liver, skeletal muscle and adipose tissue [46].

At the core of the intrinsic oscillatory circadian rhythm is a phylogenetically conserved network of transcription factors with a negative regulatory feedback cycle that lasts about 24 hours and termed the molecular clock. In mammals, the molecular cycle starts when CLOCK and BMAL1 activate *Period* (Per1, Per2, Per3) and *Cryptochrome* (Cry1, Cry2). These transcription factors form a complex and translocate to the nucleus where their interaction with CLOCK and BMAL1 negatively regulates their transcription. Similar molecular circadian clocks are found across the animal kingdom.

Physiological circadian rhythms and associated behaviors result from the integration of internal and external timing cues by a central clock which acts as a master pacemaker. In mammals, the central clock is a nucleus of neurons and glial cells in the anteroventral hypothalamus called the suprachiasmatic nucleus (SCN), while in flies, it is a network of less than 200 cells [47, 48]. Light is one of the major external cues regulating biological rhythms. As such, the SCN receives neural input from light sensitive photoreceptor cells in the retina called intrinsically photoreceptive retinal ganglion cells (ipRGCs). The SCN directly affects peripheral cells in the body through autonomic and hormonal pathways, and also indirectly by modulating body temperature and eating behavior [49]. Although the SCN is the main regulator, it is important to be reminded that almost every cell in the body contains its own molecular circadian clock, and peripheral organs (e.g., liver, kidney, lung) have as a result self-sustained circadian oscillations [49, 50].

Circadian variation in autophagy levels is well established (for a review see [7]). Already five decades ago, it was demonstrated by electron microscopy that different tissues have circadian variation in autophagy levels [51, 52]. An important study from 2011 showed that levels of the autophagy markers LC3 and p62 in mice liver varied both across the day and between the light and dark cycles [6]. The rate of LC3-I to LC3-II conversion indicated that autophagy flux peaked in the afternoon and then decreased in the dark phase. The same pattern of rhythmicity has since been found in the hippocampus [53]. Circadian changes in p62 and LC3 levels were also observed in kidney, heart, and skeletal muscle tissue [6]. Protein levels of Gabarapl1 and ULK1, but not Atg7 and Beclin1, also showed daily variations. qPCR analyses indicated oscillations during the light/dark cycle for expression of genes involved in autophagy like *Ulk1, Gabarapl1, LC3B*, and for the mitophagy receptor *Bnip3*, and also the lysosomal genes *Ctsl* and *Atp6v1d* [6]. In addition, the transcription factor C/EBP was identified as important in regulating circadian rhythms in the liver, and in linking circadian rhythms to autophagy [6]. Similarly, the transcription factors, TFEB and TFE3, which are crucial for lysosomal biogenesis and autophagy [22], display a cyclic pattern which is dependent on food intake and independent of circadian clocks [54]. Depletion of both TFEB and TFE3 in mice disrupts the autophagy rhythm. These transcriptions factors also directly regulate the expression of the key clock gene Rev-Erbα [54]. A recent study investigating the effect of the clock gene BMAL1 on autophagy has found BMAL1 to broadly influence autophagy in astrocytes. Bmal1 knock-out resulted in the accumulation of autophagosome-like structures within astrocytes. This accumulation was accompanied by increased endocytosis, lysosome-dependent protein cleavage, and the accumulation of RAB7- and LAMP1-positive cells [55].

In Drosophila, rhythmic autophagy has been observed in clock neurons, specifically PDF-expressing neurons, which produce neuropeptide-dispersing factor (PDF) and are involved in regulating circadian rhythms. These neurons include the small and large lateral ventral neurons (sLNv and lLNv), located in the accessory medulla. Autophagosome formation and processing within these neurons exhibits day-night patterns [56]. Daily changes in autophagy were shown to correlate with changes in neuronal plasticity in these circadian clock neurons, where proteins involved in membrane remodeling showed higher concentration in the morning. Moreover, Atg8-positive vesicles were found outside the sLNv terminals, suggesting a role of secretory autophagy in the regulation of the clock signaling network. These recent findings indicate that autophagy might influence the circadian pacemaker function in drosophila by remodeling terminal membranes and thereby promoting the secretion of proteins critical for clock function [56].

Dietary patterns that follow circadian rhythms also have a significant impact on autophagy. Restrictive diets aligning with the 24-hour circadian rhythm – such as limiting food intake to a 4–12-hour window – can affect the metabolism of lipids, glucose and amino acids through pathways involving AMPK, mTOR, D-β-hydroxybutyrate and neuropeptide Y, in ways that enhance autophagy. This alignment can enhance clearance through autophagy and potentially prolong lifespan [57]. A direct demonstration of the circadian clock and autophagy as key mediators of time-restricted feeding benefits comes from Drosophila [58]. Flies on an intermittent time-restricted feeding dietary regimen showed robust lifespan extension and delayed onset of aging. It has also been shown that circadian rhythm-restricted diets increase the expression of brain-derived neurotropic factors in the forebrain, which regulate autophagy and promote synaptic plasticity, as well as survival of newly formed neurons, thereby enhancing cognitive performance [59, 60]. Importantly, to realize the different health benefits of time-restricted feeding, it appears that the feeding period must occur during the active phase of the circadian cycle [58].

Further links between autophagy and circadian regulation include molecules such as SIRT1, a NAD+ dependent histone deacetylase which acts on different clock proteins [61]. The energy sensor AMPK phosphorylates some clock genes [62] and mTOR has been linked to the regulation of both central and peripheral clocks [63].

## Sleep – definitions and functions

Sleep can be defined as a natural, rapidly reversible and recurring state of reduced responsiveness, consciousness, motor and metabolic activity [64, 65]. As it is quickly reversible, natural and non-pathological, it can be separated from other states of reduced consciousness such as hibernation, anesthesia and coma [66]. In addition to its behavioral and physiological characteristics, sleep is further defined by its regulation through the circadian clock and a homeostatic mechanism that builds sleep pressure during wakefulness [67]. This dual regulation ensures the periodic recurrence of sleep on a daily rhythm in natural conditions, while also explaining variability in response to unnatural disruptions such as jet lag or shiftwork. As stated at the start of this review, sleep is vital for normal functioning. Sleep disturbance can impact health in numerous ways, from impairing emotional and cognitive functions to potentially leading to the emergence or exacerbation of some pathologic comorbidities [68–70].

Still, the functions of sleep are not fully understood, but suggested roles include energy conservation, tissue repair, thermoregulation, metabolic functions, immune functions and memory consolidation [65, 71].

**Figure 2:**
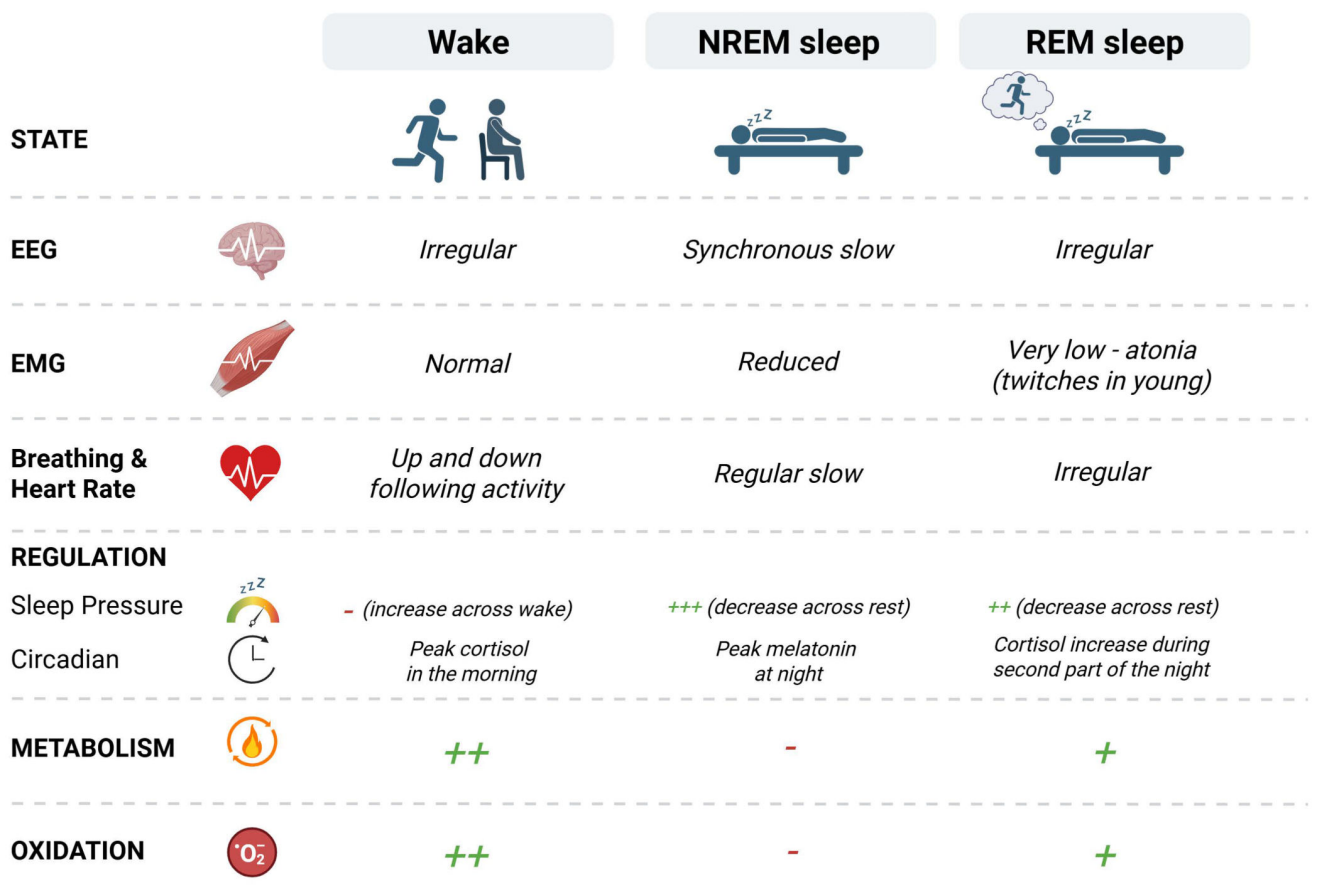
Overview of multimodal physiological and regulatory differences across wakefulness, NREM sleep, and REM sleep. Shown is the characteristic EGG activity, oscillatory patterns, muscle activity (EMG), breathing and heart rate, regulatory components, metabolism and oxidative processes across states.

Mammalian sleep consists of two alternating physiological states: rapid eye movement sleep (REM, also known as paradoxical sleep) and non-REM sleep (NREM) (Fig. 2). REM sleep is characterized by desynchronized, fast brain waves, rapid eye movements, low skeletal muscle tone with intermittent twitches, irregular respiration & temperature, as well as an elevated arousal threshold. It is associated with vivid dreaming in humans [72]. On the other hand, NREM sleep presents coordinated slow brain waves, slow regular respiratory & cardiac rhythms, low temperature & metabolism [73]. Experimentally, REM and NREM sleep are distinguished from wake states in vertebrates based on cortical (EEG, electroencephalogram) activity in combination with either muscular activity (EMG, electromyogram) or eye movements (EOG, electrooculography).

Across a rest period, REM and NREM sleep states alternate. In human adults, a typical night of sleep comprises 4–5 cycles of about 90 mins with an abundance of slow wave sleep in the early night, while REM sleep dominates late sleep [65, 74]. Sleep amounts and architectures greatly differ depending on species, age, and health status. Carnivores such as lions and brown bats can sleep up to 20 hours per day, hinting at greater sleep needs linked to metabolic functions. At the other end of the spectrum, big prey such as giraffes and horses sleep as little as 2–3 hours a day [75]. Both total sleep and REM sleep amounts decrease drastically across the development of altricial mammals (i.e., born with an immature brain). Human infants sleep about 16 hours per day, 50% of it being REM sleep. As they grow, sleep and REM sleep amounts sharply decline to reach about 8 hours (25% of REM sleep) in healthy adults. Sleep quality can vary across adulthood. Self-reports show a steady decrease in sleep duration until retirement age when it may increase again [76], yet it is difficult to assess how life and work circumstances influence this data. A consensus is that in experimental conditions, sleep amounts in younger adults are noticeably higher than those recorded in elderly which exhibit on average 6 hours of sleep (15% REM) [76, 77]. Furthermore, at least half of the elderly population are reported as suffering from sleep disorders [78, 79].

## Sleep regulation

Sleep is regulated by at least four key mechanisms: 1) a *circadian* pacemaker, consisting of central and peripheral clocks; 2) a *homeostatic* sleep pressure, which reflects the body’s increasing need for sleep with prolonged wakefulness; 3) interoceptive modulation by internal physiological states, such as hunger, thirst, and thermoregulation, that can override the sleep-wake pattern based on competing homeostatic needs; 4) exteroceptive modulation by environmental stimuli including light, noise, social interactions and other forms of sensory input that can impact sleep by masking internal rhythms or acutely promoting wakefulness. Light in particular has both clock-dependent and independent effects on sleep regulation [4, 80]. These different mechanisms interplay at the level of the central nervous system through a complex neural network of reciprocal inhibition between wake promoting arousal neuron circuits and sleep-inducing ones (Scammel 2017). We provide a brief overview of the sleep regulatory mechanisms below; however one should note that they are quite complex, and we recommend the following reviews for in-depth description of these processes, see [10, 74, 81–84].

In brief, pressure for sleep builds up as one keeps awake, through varied homeostatic processes [85]. Sleep/wake transitions in mammals are regulated by a complex network of interconnected brain nuclei, mainly located in the brain stem, the hypothalamus and the forebrain, either promoting wakefulness, NREM or REM sleep [84]. Circadian rhythms modulate sleep-wake regulations according to the internal clock and external synchronizations with the daily rhythms [10, 86]. Wakefulness is maintained when the circadian arousal drive is high, the sleep pressure is low, and/or through behavioral needs (e.g., stress, motivation, cognitive or motor demands). When the sleep pressure “wins”, one falls into NREM sleep. REM-promoting circuits inhibit both the wake- and the NREM-promoting circuits, as well as muscle tone [84]. Many postulate that the reciprocal inhibition in the central nervous system between wake- & sleep-promoting circuits leads to a so-called “flip-flop mechanism”, ensuring that an individual is either asleep or awake [87, 88]. Yet, some evidence points to the existence of local brain states, with neuron populations exhibiting “sleep patterns” while an individual is awake [89, 90]. This could widen the definition of sleep beyond a global state - with implications for local sleep homeostatic and cellular mechanisms such as autophagy, yet we still know very little on this subject.

While circadian rhythms are crucial regulators of sleep, they should not be equated with sleep itself [91]. The interaction between sleep and circadian clocks is not fully understood. Although earlier studies suggested that molecular clocks directly influence the timing of sleep [92], more recent studies indicate that clock mutations also affect total sleep duration and sleep quality [81, 93, 94]. As such, the sleep/wake regulation has been modelled as a two-process mechanism composed of a circadian pacemaker (Process C) and a homeostatic sleep regulator (Process S) [80]. The homeostatic sleep drive is mainly mediated by sleep-promoting substances called somnogens, whose concentration in the brain (or the blood flow) rise during wakefulness and induce drowsiness to promote the transition to sleep [91]. Among prominent somnogens, one can find prostaglandin, cytokines (mediators of inflammation) and adenosine (whose antagonist is caffeine). On the other hand, the circadian pacemaker action is to consolidate sleep and wake into larger blocks while promoting sleep blocks to be predominant during the dark (diurnal species, e.g., human) or the light period (nocturnal species, e.g., bats). This effect is mainly mediated via neuroendocrine pathways, following the combined action of an endogenous 24h-periodic rhythm with the propensity to shift the onset of that rhythm in response to both environmental (e.g., light, temperature) and behavioral (e.g., feeding, exercise) cues [86, 95]. Similarly to the circadian regulation of autophagy, the key circadian regulator of mammal sleep is the SCN of the hypothalamus. Lesions of the SCN lead to a disturbed wake/sleep pattern with unconsolidated random bouts of sleep unrelated to the light–dark cycle, while only mildly affecting total sleep amounts [96]. A main player in circadian sleep regulation in diurnal species is the neurohormone melatonin whose production by the pineal gland (or epiphysis cerebri) is tightly regulated to the light activation of the SCN neurons [97]. This is mostly true in diurnal species, but recent evidence shows that melatonin’s role in the timing of sleep onset is conserved in nocturnal mice [98]. Prescription/use of melatonin supplements has escalated in the last years, as recent changes to human living environments, work or social schedules and patterns of light exposure are increasingly misaligned with our endogenous rhythm. Interestingly, the role of melatonin differs across species. While it is associated with sleep in humans, it promotes wakefulness in some nocturnal animals [99]. Beyond its role in sleep, melatonin has broad physiological effects, including the modulation of metabolism, oxidation, and autophagy mechanisms. For example, it can induce or maintain autophagy under physiological conditions while also preventing excessive autophagy during cellular stress [100]. Acknowledging the dual role of melatonin is therefore important when investigating the complex relationship between autophagy and sleep, as its effects may vary based on context and species. Furthermore, endocrinal-sleep regulation mechanisms are greatly perturbated during hormonal shifting paradigm life periods such as puberty, pregnancy, and menopause. As such, lifespan and gender perspectives are essential to consider when interpreting studies.

In drosophila, sleep is defined from a behavioral perspective where prolonged periods of immobility are used as a proxy for sleep [101, 102]. Typically, 5 minutes of inactivity is defined as sleep, since the flies then exhibit an increased threshold for arousal. Laboratory flies are most active in the light-to-dark and dark-to-light transitions and sleep mainly in the middle of the night and the middle of the day, although with some gender-specific variations [103]. Sleep in drosophila is naturally influenced by environmental factors such as temperature, food availability, socialization and success in courtship [104].

## Metabolic changes during sleep

There are a variety of metabolic changes occurring during the transition from wake to sleep. It is estimated that sleep decreases the total metabolic rate by about 15% [105].

Cortisol and growth hormone (GH) are two important hormones which display different patterns during the sleep/wake-cycle. Growth hormone shows a pulsatile release which is highest during slow wave sleep [106]. Although this pulsatile release is closely tied to the sleep-wake cycle, its regulation is primarily circadian. Cortisol release, in contrast, follows a clear circadian rhythm. Its nadir occurs around midnight, after which it increases, reaching its peak in the morning for humans. In nocturnal animals, cortisol levels are highest in the early part of the night [107]. Sleep-wake also regulates cortisol levels and slow wave sleep has an inhibiting effect on cortisol secretion [108].

While an individual is engaged in an active behavior, their brain primarily uses glucose as energy substrate through aerobic glycolysis, which can thus be regarded as a hallmark of wakeful state. A high glycolytic metabolism during wakefulness results in a net lactate production [109]. As brain lactate levels decrease quickly, this is regarded as the only good metabolic biomarker of sleep [110]. Whereas sleep is not associated with increased blood glucose levels, it is linked with elevated brain glucose levels, attributed to a reduced cerebral glucose metabolism. On the other hand, recent evidence showed that peripheral glucose concentrations in the interstitial tissue can be regulated by neural oscillations in the hippocampus specifically associated to rest periods [111].

During sleep, there is a general metabolic shift in the brain and the rest of the body from glucose utilization to increased oxidation of fatty acids and ketone bodies [109]. Levels of the two main ketone bodies, B-hydroxy-butarate and acetoacetate, are upregulated during sleep if a time restricted feeding protocol is used [109, 112]. Another interesting change occurs in the first hours after falling asleep, when there is a surge in ATP-levels in “wake-active”, but not in “sleep-active”, brain regions in the rat. This surge is followed by a concomitant reduction in p-AMPK [113].

## Metabolic waste clearance during sleep

The brain has a high metabolic rate and is highly susceptible to toxic metabolic byproducts. Waste clearance is consequently a vital process for maintaining neuronal health. Surprisingly, the brain does not have a conventional lymphatic system but relies on the circulation of cerebrospinal fluid (CSF) interchanging with interstitial fluid (ISF), facilitated by aquaporin-4 (AQP4) channels located on astrocytic endfeet, to eliminate metabolic byproducts [114]. This system is referred to as the glymphatic system. The function of the glymphatic system has been especially studied in the context of neurodegenerative diseases such as Alzheimer’s disease, for which waste clearance is a potential therapeutical target. Notably, toxic Aβ protein has been observed to be higher during wakefulness. This was initially attributed to the increased metabolic activity and subsequent waste accumulation during active behavior of healthy human subjects and animal models of Alzheimer’s disease [115, 116]. However, the focus has lately shifted towards brain clearance which increases drastically during sleep in healthy subjects but fails to do so in non-healthy [117]. It was reported that the volume of interstitial space increases by 60% during sleep, thereby increasing the convective exchange of CSF with ISF, which consequently increases the rate of waste clearance [118, 119]. Furthermore, recent studies report sleep cycle-dependent perivascular dynamics in the glymphatic system, hinting at different contribution between REM and NREM sleep in brain clearance [120]. This function appears conserved since a discrete sleep stage has been identified in Drosophila where the proboscis is repeatedly extended and retracted, resulting in increased waste clearance [121]. Both in flies and mammals, sleep deprivation and sleep loss seems to impair such clearance mechanisms while subsequent sleep rebound cannot fully compensate for the previous lack of clearance [119, 121, 122]. Although multiple studies have reported an increase in clearance during sleep, one should note that some studies have challenged this result [123]. Some suggested that the increase in clearance might be influenced by the circadian rhythm, which modulates AQP4, rather than sleep [124]. Further research is needed to clarify the influence of sleep and circadian rhythm on waste clearance in the brain.

## Oxidative stress and sleep

Oxidative stress arises when the production of reactive oxygen species (ROS) exceeds the antioxidant defense capacity. Such imbalance leads to structural damage at the cellular level as proteins, lipids and nucleic acids are vulnerable to oxidation. Wakefulness poses an oxidative challenge due to increased mitochondrial ROS production [125–127]. In contrast, mitochondrial respiration - and consequently ROS production - is lower during NREM sleep [128]. The reduction in mitochondrial activity likely explains why oxidative stress is lower during NREM sleep. Additionally, it has been suggested that sleep not only minimizes oxidative stress but also facilitates the removal of accumulated free radicals [129]. In the brain, uridine and glutathione may play a major role in the facilitation of oxidative detoxification by enhancing GABAergic transmission and inhibiting glutamatergic transmission [130].

Several sleep deprivation and sleep loss studies reported reduced levels of key components of the body’s antioxidant defense system, such as glutathione, superoxide dismutase and catalase, both in brain and liver tissue [126, 127, 131]. However, other studies observed no change in antioxidant activity or oxidative damage in the brain, liver, and skeletal muscle [132, 133]. A recent systematic review concluded that evidence supporting the presence of oxidative stress after sleep deprivation outnumbers the reports against changes in oxidative stress parameters [134]. Amongst the reports supporting this conclusion are studies demonstrating that a lack of sleep increases the sensitivity to oxidative stress in drosophila [125] as well as accumulation of ROS in the gut of drosophila and mice, which was identified as the driving factor of the effect of sleep loss on longevity [135]. An increase in sleep duration, in contrast, has been shown to increase the resistance to oxidative stress as preventing the accumulation of ROS alone allowed the survival of sleep deprived drosophila [125].

Emerging research also suggests a bidirectional relationship between oxidative stress and sleep. Sleep loss has been found to increase mitochondrial ROS in sleep-regulating neurons in drosophila. This increase in oxidative stress is promoting sleep through the modulation of ion channel activity through redox-sensitive mechanisms [136]. Furthermore, reducing oxidative stress through the overexpression of antioxidant genes in neurons has been shown to reduce sleep time in drosophila [125]. ROS may act as regulator of redox-sensitive transcriptional factors and other effectors which, in turn, regulate sleep [137].

While the exact relationship between sleep and oxidative stress remains unclear, current evidence largely supports an essential role of sleep in maintaining cellular homeostasis by mitigating oxidative damage. Beyond this perspective, recent findings suggest oxidative stress to be not only a consequence of wakefulness but also an active driver of metabolic adaptation across the sleep-wake cycle. More specifically, it has been shown that the mitochondrial oxidation in neurons during wakefulness leads to the formation and transfer of lipid droplets carrying oxidative byproducts to glial cells. The clearance and recovery of mitochondrial function then requires sleep [138]. Together, the current literature suggests a coordinated interplay between oxidative stress, energy metabolism and sleep, essential for cellular maintenance and health.

## Autophagy and physiological sleep

At the heart of this review is a line of reasoning based on the glymphatic hypothesis that postulates that sleep is essential to clear waste in the extracellular space of the brain. Following this hypothesis, could autophagy “clean the cells from the inside” specifically during the off-line and hypometabolic state that is sleep – in concert with or independently of the glymphatic system? Although few studies have directly examined the differences in autophagy between physiological sleep and wakefulness, several have explored how autophagy flux varies throughout the day and night, revealing a strong circadian regulation [53, 139, 140]. A key study that directly assessed the relationship between sleep and autophagy was performed in Drosophila [141]. They observed a higher number of autophagosomes in the early night compared to the morning, suggesting a role for sleep in autophagy regulation. Furthermore, they observed an accumulation of autophagosomes in the morning following sleep deprivation, while an increase of sleep through administration of gaboxadol resulted in a reduction of autophagosomes. These findings indicate that the reduction of autophagosomes during sleep was due to sleep itself rather than merely a circadian rhythm-driven mechanism [141].

If autophagy occurs preferentially during sleep, one can easily imagine that components regulating or executing autophagy may modulate sleep onset or sleep architecture. To investigate this hypothesis, one study silenced positive (Rheb) or negative (TSC1) regulators of TOR, TOR itself, or the autophagy core component Atg5 in Drosophila [142]. They observed an increase in sleep time when TOR or Atg7 was knocked down, whereas the depletion of Atg5, Rheb or TSC1 led to no sleep disturbance. In addition, the Sehgal lab systematically depleted genes related to autophagy in adult Drosophila, either in the whole organism or specifically in neurons. They found that neural depletion of Atg1 or Atg10 lead to a marked increase in total sleep amount [141]. Moreover, sleep modifications were observed upon depletion of Atg1, Atg7, Atg12, Atg8b, Atg14, or proteins involved in autophagosome maturation in the entire adult fly. Specifically, flies depleted of Atg1 in all tissues during adulthood showed increased total sleep time and decreased sleep latency.

Further evidence linking sleep and autophagy comes from a *Drosophila* sleep-mutant named *argus (aus)* [141, 143]. This mutant exhibited concomitant (i) accumulation of autophagosomes and (ii) reduced total sleep time. Sleep loss in the *aus* mutant flies was linked with an inability to sustain sleep, while circadian rhythms remained intact in most animals. More evidence comes from a mutant for the gene *blue cheese 58* (*bchs* – the drosophila homolog of human Alfy) which is involved in autophagy. Defects in this gene can lead to impaired autophagy and neurodegeneration [144]. Contrary to the *aus*-mutant, the *bchs*-mutants displayed an increase in total sleep time and a decreased accumulation of autophagosomes. Combined together, these observations led the authors to hypothesize that autophagosomes accumulation influence sleep behaviors [141].

In addition to this evidence linking general autophagy to sleep, a more specific function of mitophagy (selective degradation of mitochondria through autophagy) has been proposed to be part of a sleep-regulated mitochondrial lipid metabolic cycle across neurons and glia [138]. The authors found that neurons protect themselves from accumulating oxidative mitochondrial damage during wake through mitophagy and through the transfer of damage to glia in the form of lipids. The glia then requires a full night of sleep to clear the lipids and recover from the mitochondrial oxidation. Sleep is also required for maximal mitophagy in the neurons [138].

## Sleep manipulation models

Sleep manipulations models may aim at increasing or decreasing sleep quality and/or quantity, or at dysregulating the alignment between sleep and circadian rhythms. They can be divided into: (i) total sleep deprivation (24 hours), (ii) partial sleep restriction (N hours out of 24 hours), (iii) sleep fragmentation (unconsolidated fragmented sleep bouts spread over 24 hours without altering total sleep time) and (iv) sleep quality manipulation (e.g., targeted manipulation to specific sleep states such as REM) [145]. The latter two are sometimes referred to as “sleep architecture” manipulations. When evaluating the effect of sleep manipulation, one should consider whether a sleep rebound occurs (or not) before measures are taken (i.e., extra amounts of time that a subject spends asleep following a restriction protocol compared to a control subject – consequences of sleep homeostatic regulation).

There are many methods to manipulate sleep, following the vast complexity of the sleep regulation systems [146, 147]. A list of all sleep manipulation models is well beyond the scope of this review. Table 2 shows some of the main rodent and drosophila models that have been used in autophagy-related sleep studies.

## Does sleep impairment increase autophagy?

**Figure 3:**
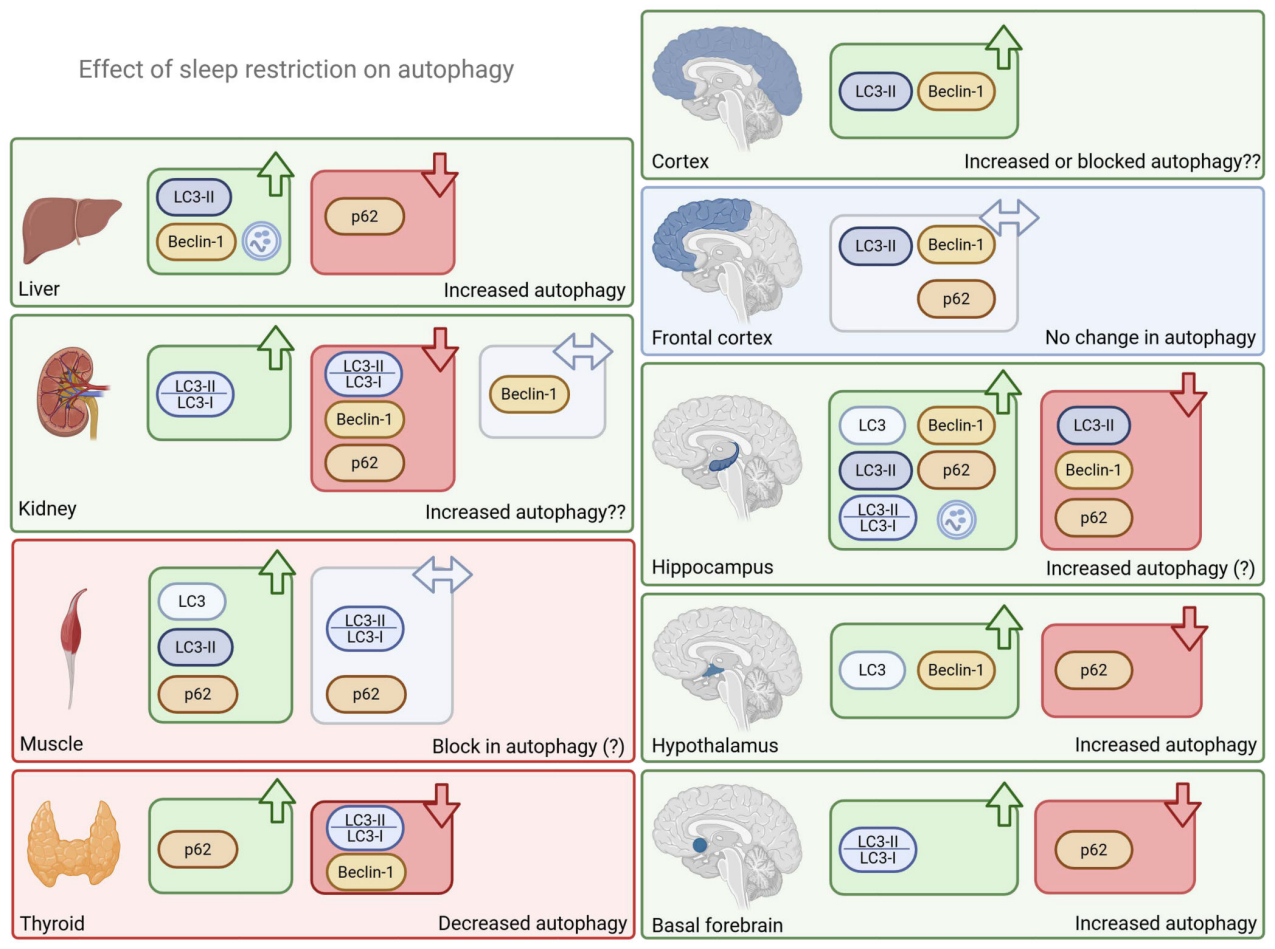
Effects of sleep restriction on autophagy. The current evidence for effects on autophagy of sleep restriction in brain and peripheral tissues are summarized here. The evidence is mainly from rodent models and largely based on immunoblotting for the indicated targets, although electron microscopy or immunohistochemistry have also been used. Note that proper flux assays have not been performed in the majority of the studies, therefore all conclusions on overall autophagy change are tentative.

The vast majority of studies to date have reached the same conclusion; methods used to impair sleep result in autophagy activation (Fig. 3). Why? The logical interpretation of this result seems straightforward. Disrupting sleep clearly represents a stress both at the organism and probably also at the cellular level. Autophagy can be induced because of a variety of cellular stressors including deprivation of growth factors or nutrients, DNA damage, hypoxia, ROS, protein aggregates or damaged organelles [148]. Partial sleep deprivation has been known to induce DNA-damage in older adults [149] and, as discussed earlier, research suggests sleep loss can lead to accumulation of ROS and oxidative damage [135] as well as attenuated mTORC1 signaling and protein synthesis [150]. Thus, sleep loss or fragmentation in fact induces several cellular stressors and decreases mTORC1 activity, which all can be plausible biological mechanisms resulting in increased autophagy.

Yet, we still do not have experimental evidence clearly supporting these mechanistic interpretations and further experiments are required. The current methods employed to address these questions are limited in their scope. Most studies only used western blotting to measure autophagy. This is problematic given that autophagy is a dynamic process, whereas western blotting gives a snapshot of protein levels frozen in time. This limitation can partly be addressed by sampling at multiple time-points (although from different animals) and by including lysosomal blockade to enable assessment of autophagy flux. Furthermore, most studies assessing autophagy impacts of sleep impairment measured LC3-II/LC3-I-ratio and p62 levels, with the limitation whether this reflects actual changes in autophagy flux as discussed in the section about autophagy methods.

## Different effect of sleep restriction on different organs?

Interestingly, most studies that investigated LC3 levels upon sleep restriction found an increase in the LC3-II/LC3-I ratio, LC3-I levels or total LC3 levels (Fig. 3) [151–157]. These studies typically performed full or partial sleep deprivation using either the multi-platform water environment method or tactile stimulation in adult rodent subjects over 2–5 days and assessed autophagy in the hippocampus. Surprisingly, when autophagy was measured after longer periods of sleep restriction, opposing result were reported across brain regions: an increase in LC3-II protein levels and number of LC3B+ cells was reported in the cortex following 2 months of sleep restriction [158], while a decrease in LC3-II, LC3-II/I and Beclin1 was found after 20 days of sleep restriction in the hippocampus [159]. Interestingly, rebound sleep after 21 days of sleep fragmentation also showed a disruption of the circadian rhythmicity of autophagy in the hippocampus [53]. A study assessing autophagy in rat thyroid tissue upon sleep restriction also reported decreased levels of LC3 and Beclin1, along with increased p62 levels suggestive of decreased or inhibited autophagy [160]. Similarly, a decrease in Beclin1 has also been reported in kidney tissue after 28 days of sleep restriction [161]. This could potentially reflect tissue-specific mechanisms – both in short- and long-term responses – or reflect variations in sleep restriction/deprivation protocols. In contrast, increased levels of LC3-II and Beclin1 and decreased levels of p62 were observed in liver tissue from rats subjected to 3 weeks of sleep restriction, indicating increased autophagy [162]. These apparently contradictory results could be the consequence of metabolic stress that would lead to an abundance of lysosomes in liver cells and the liver's high capacity for autophagy [163]. One possible interpretation is that sleep restriction drives sustained demand for autophagy, but that the availability of one or more critical autophagy proteins may be exhausted if sleep restriction is sustained for too long. This could eventually cause a flip from a surplus number of autophagosomes during short sleep restriction to a deficit during long-term sleep restriction. This possibility is consistent with the paradoxical longer-term sleep restriction results reported in the liver [162], since the higher starting capacity for autophagy in the liver may take much longer to deplete. In any case, when assessing effect on autophagy, one should aim to control whether apparently divergent effects between organs/tissues could result from an organ-specific interaction of a specific sleep restriction/deprivation protocol with metabolic or oxidative stress.

## Tissue-specific effect of sleep restriction between brain regions?

When it comes to the effects of sleep restriction on levels of the autophagy cargo receptor p62, studies have shown highly contrasting results (Fig. 3), even when considering the same brain tissues (i.e., hippocampus). Such opposite results may be explained by different use of lysis buffer that have been demonstrated to influence the p62 levels detected by western blot [40, 164]. Several of these studies used both high and low p62 levels as evidence for increased or excessive autophagy upon sleep impairment, without proper flux assays. Despite such protocol limitations, the overall conclusions from these studies concurred based on the increased LC3 (and Beclin1) levels: sleep deprivation leads to increased or excessive autophagy. Interestingly, one study comparing different brain tissues found that sleep fragmentation induced increased p62 levels in the striatum, decreased levels in the hippocampus, whereas levels remained unchanged in the frontal cortex [154]. Another study reported decreased p62 levels in the basal forebrain [165]. This indicates that different brain regions exhibit tissue-specific differences in autophagy response to disrupted sleep. Adding to this, Guo and coworkers reported that sleep fragmentation increases levels of corticotropin-releasing hormone (CRH) in the hippocampus – but not in the striatum or the prefrontal cortex – and further demonstrated that CRH dysregulated autophagy in vitro [166]. However, the mechanism and functional implications of these differences is not yet understood. Could it be linked to differences in sleep protocols that stimulate/challenge one brain region (sensory vs motor stimulation) rather than the other? Is this dependent on plastic mechanisms? – The hippocampus is one of the most plastic regions of the brain and it would respond to new experiences in a contrasted manner from other regions. Collectively, these results highlight the need for future studies with better control of sleep-deprivation conditions to further investigate the tissue-specificity of sleep restriction and deprivation on autophagy.

## Is the mTOR pathway involved?

Some sleep deprivation studies included the investigation of the PI3K-Akt-mTOR pathway, which regulates cell survival, autophagy and apoptosis. Most found either unchanged or reduced levels of the proteins in this pathway. It has been suggested that mTORC1 signaling and protein synthesis are reduced in the hippocampus of sleep deprived mice, thus leading to the impairment of the protein synthesis needed for learning and cognition [150]. Interestingly, sleep deprivation-induced aberrant autophagy in hippocampal microglia and associated cognitive impairments could be prevented by silencing S100A8, which activates the PI3K-AKT pathway [167].

In a study focusing on the rat gastrocnemius muscle, increased levels of p-mTOR, p4E-BP1 and 4E-BP1 were reported upon sleep deprivation [168]. However, the nutritional status of the animals at the time of sacrifice was not documented, which could have influenced these results. Other studies of skeletal muscle did not detect any change in protein levels of Akt or mTOR [169], while another reported local decreased p-Akt in temporal, but not masseter muscle tissue upon sleep deprivation [170]. Collectively this evidence suggests that the strong catabolic observed in the hippocampus is not as prominent in skeletal muscle.

## Is autophagy induced by sleep-disruption beneficial to health?

A central question to many of the studies we reviewed was whether the apparent increased autophagy upon sleep disruption is beneficial or detrimental to health? Several authors claim that sleep deprivation led to “excessive autophagy”. Some of these studies investigated treatment with drugs that may increase wakefulness such as modafinil [153], or induce anesthesia/drowsiness such as propofol [152]. Other investigate the effect of diverse treatments that impact brain activity: SLSP [155, 156], H2S [151, 171], and theta burst stimulation using transcranial magnetic stimulation (TMS) [172]. Collectively, these treatments show a link between attenuated autophagy levels, reduced cognitive impairment and protection from cell death. The effect of these treatments in inhibiting autophagy was further confirmed with in vitro experiments, although for H2S both induction and suppression of autophagy has been reported [173]. A conclusion from these studies is that autophagy inhibiting treatments may mitigate adverse effects of sleep impairment. Yet the effects shown were only correlative but not causal. As such, further experiments are needed to demonstrate that a treatment aimed at decreasing the autophagy response could protect the sleep deprived brain against aberrant cell death and cognitive impairment. Furthermore, the drugs used in these studies had many side effects and more targeted manipulation are needed.

In contrast with these results, the autophagy-inducing drug rapamycin was also shown to protect the kidney against damages induced by sleep-deprivation through the mTORC1 pathway [174]. Interestingly, opposite effects were observed in the hippocampus, where electropuncture suppressed autophagy, as measured by LC3-II/-I ratios, via the mTOR signaling pathway [175]. This dichotomy between apparently contradicting results yet again highlights the tissue-specific roles of pathways involved in autophagy regulation during sleep, as mTOR activation potentially offers neuroprotection in the brain, while mTOR inhibition may support resilience in the kidney.

An important controversial subject that needs to be addressed also in the context of sleep-deprivation is the following: Do cells actually die via autophagy? Or is autophagy just a bystander process? Or is autophagy trying to prevent further damage? Autophagic cell death has historically been regarded as a type of cell death in addition to apoptosis and necrosis. A newer term is “autosis”, a type of autophagy dependent cell death [176]. Autosis can be induced by starvation, autophagy inducing peptides and hypoxia-ischemia, and is characterized by the disappearance of the ER and the swelling of the perinuclear space [177]. There is also crosstalk between autophagy and apoptotic machinery, linking autophagy to another type of cell death [178]. Therefore, it is certainly possible and plausible that excessive autophagy contributes to the adverse effects on health linked to sleep disturbances. To test this hypothesis, future studies should use a combination of genetic and pharmacological manipulations of autophagy with sleep impairment protocols. Such comprehensive approaches could help to better delineate the specific role of autophagy during sleep or after sleep disturbance. In other words, they would clarify whether excessive autophagy is harmful and mediates some of the negative outcomes associated with sleep disruption or on the contrary helpful to alleviate them.

## Does autophagy impairment impact sleep?

A few studies have manipulated autophagy and measured its impact on sleep. A higher total sleep amount was observed when autophagy was inhibited by knocking down Atg1, Atg7 or Atg8 [141]. Interestingly, depletion of TOR and overexpression of Atg8a in a Huntington’s disease drosophila model [179], which should result in autophagy induction, also increased sleep time. Why would seemingly opposite autophagy manipulations both result in increasing sleep? Insights from Drosophila research might provide some clues there. We already outlined a study for which both *bchs* mutants and *aus* mutants exhibited disrupted autophagy function, yet with opposite effects on autophagosome numbers (reduced in *bchs* and decreased in *aus*) and sleep (increased in *bchs* and decreased in *aus*). This led us to hypothesize that autophagosome number regulation is a potential central mechanism by which autophagy may modulate sleep. We postulate that the depletion of TOR or different Atgs may reduce autophagosome number, either by increasing autophagosome turnover (such as in the absence of TOR) or by preventing autophagosome formation (when Atgs are downregulated). This could explain the similarity in sleep phenotype in apparently opposite manipulations. A possible hypothesis is that impaired autophagy could impact the levels of somnogens and as such modulate the homeostatic sleep pressure.

## On the influence of the sleep manipulation protocols

The most frequently used sleep manipulation method combined with autophagy assays has so far been the MMPM (modified multiple platforms method) during which rodents are keeping awake in order not to fall in the water. The main weakness of this method is that it induces a stress response that may directly induce autophagy, thus confounding the interpretation of the relationship between sleep manipulation and autophagy. A study that specifically controlled the stress response in MMPM protocols showed that mice that spent 14 hours daily on the platforms exhibited stress after 5 days [180]. By 21 days, animals showed signs of anxiety, downregulation of NMDA receptor gene expression and morphological degeneration in the hippocampus [180]. Corticosterone levels were significantly increased after 5 and 14 days, in agreement with previous studies [181]. Additional potential cofounding factors in MMPM are the monotony of the task, the fatigue from long-term standing and the lack of environmental changes. These factors may contribute to increase the stress response [180]. Another study used surgery in which the tympanic membrane was penetrated to induce vestibular damage [182]. This is an invasive and stress inducing technique. The conclusions pertaining to autophagy in this context may merit to be revisited with more gentle methods for sleep deprivation.

Unfortunately, most studies assessing the effects of sleep disruption on autophagy do not report measurements of stress responses. However, the few studies that did it reported an increase in serum corticosterone [157, 168, 169, 183]. In contrast, a study using a sleep fragmentation protocol via tactile stimulation for five days, did not detect elevated mRNA levels of CRFR1 or CRFR2 in the striatum, cortex or hippocampus [154]. At the protein level, CRFR2, but not CRFR1, was increased in the striatum of sleep-fragmented mice, whereas neither were increased in cortex or hippocampus. We advocate that (i) all studies testing the impact of sleep disturbance should systematically include stress levels measurement and that (ii) methods with lower stress response, should be preferentially chosen.

Similar recommendations should be applied to all species. For example, drosophila sleep deprivation protocols typically use locomotor tubes in which the flies are shaken every 20 seconds [141] or placed on a vibrating platform [184]. Such procedures are likely to induce stress and more gentle sleep deprivation (e.g., rotation of the tube housing the fly only upon a certain period of inactivity [185]) should be privileged to avoid confounding effects on autophagy. Sleep impairment in itself has been shown to increase stress through the hypothalamic-pituitary-adrenal (HPA) axis [186] and stress response genes have been shown to be protective against sleep deprivation effects [187]. Furthermore, there is in vitro evidence that the HPA axis can impair autophagy signaling [188]. As such, measure of stress response and the HPA axis are crucial to the interpretation of sleep deprivation effects on autophagy.

## Other cofounder factors besides stress

Several studies have measured autophagy in models of sleep apnea [189–194]. As hypoxia is a known inducer of autophagy [195], once again these studies are difficult to interpret. One cannot easily determine whether the observed autophagy phenotypes are due to sleep disturbance, hypoxia, or a combination of the two. Consequently, sleep apnea models should be avoided to test the direct effects of sleep impairments on autophagy.

Finally, certain pharmacological treatments such as melatonin have been used in sleep studies assessing autophagy [157, 183]. However, pharmacological treatments may have broad effects not limited to sleep. For example, melatonin is a neurohormone regulating the onset of several circadian functions, as well as modulating metabolism and oxidation, as discussed earlier. As such, it may influence autophagy by mechanisms unrelated to sleep. This makes these studies challenging to interpret as it is unclear whether the observed effects are due to the sleep manipulation or rather the broad direct or indirect effects of melatonin itself.

## New opportunities for better controlled sleep manipulation

Emerging technologies are creating new opportunities for sleep manipulation. These include optogenetics, chemogenetics and gene editing/modifying techniques, which have rapidly expanded knowledge in the field of neuroscience. Optogenetics uses light to control the firing of specific neurons allowing precise control of neuronal activity targeting specific groups of neurons or circuits [196]. With the stimulation of sleep-regulating regions, either by activating or inhibiting targeted neurons, optogenetics could be useful for studying autophagy. Drosophila transgenic animals expressing red-shifted channelrhodopsin variants in selected neurons are a useful tool [197]. In rodents optogenetics is still invasive, involving the insertion of electrodes through the skull of the animal, which can cause damage and stress response, although less invasive optogenetics methods are emerging [198]. Chemogenetics offers remote control over neural circuits or groups of cells through systemic injection or microinfusion of a substance that will activate engineered receptors. This method is less invasive than optogenetics, but the temporal resolution is lower [199, 200] and the designer drug may not be as inert as claimed [201]. Another alternative is thermogenetics, such as transgenic Drosophila expressing the temperature-sensitive cation channel TrpA1 in selected neurons. This offers much better temporal control than chemogenetics and has been used in sleep- or wake-promoting neurons [202] to study for instance ROS generation upon severe sleep loss [135]. A drawback of thermogenetics in the context of autophagy would be that high temperatures would likely impact autophagy along with many other intracellular processes. Finally, the use of genetically modified animals is playing an increasingly important part of sleep research. Gene editing tools, such as CRISPR/Cas9, are expanding rapidly and will be very useful in elucidating the mechanisms of sleep in the coming years [203].

## Can results from animal models be broadened to the autophagy sleep function in humans?

We could only find one study that tested the effect of sleep restriction on autophagy in humans [204]. Participants were allowed 4 hours of sleep for 5 consecutive days, and autophagy levels were measured from vastus lateralis muscle biopsies. The authors reported a trend in the data for reduced p62 transcription and reduced LC3-II/I protein levels, that did not reach statistical significance. One can speculate that this study was underpowered because of a too low number of participants relative to a mild effect on a highly variable background.

The use of animal models is often necessary to specifically manipulate and monitor both autophagy and sleep mechanisms. However, it is essential to understand how sleep in the chosen model species may differ from human. For example, laboratory rodents sleep about 12 hours per day [205]. They are nocturnal, yet they may have sleep episodes during the dark period and awake activity during the light period (20–30%) [145]. Rodents are polyphasic sleepers, exhibiting many brief sleep-wake episodes within 24 hours. Their sleep architecture differs significantly from that of humans. While humans typically experience 4-5 consolidated ultradian cycles per night, of which each consists of structured NREM to REM progression, rodents show more fragmented and frequent state transition within, but also across, shorter sleep episodes. Despite these differences, rodent models are widely used and accepted as valid models in sleep research but one should be aware of these differences when interpreting results [145].

Some studies have used *Drosophila* to assess connections between autophagy and sleep. The primary advantages are the available genetic tools and their suitability for rapidly generating large amounts of data. For the last 20 years, the fruit fly has been increasingly used in sleep research, with findings suggesting that many sleep characteristics are comparable to other species [206]. Our opinion is that we should aim as a community to test autophagy hypotheses in multiple model species allowing true comparisons between mechanisms and divergent sleep architectures, as well as sleep restriction and deprivation methods. Additionally, we advocate that some autophagy studies should adopt a translational perspective. For this to succeed, it will be essential to foster close collaborations and integrated research efforts between clinical settings and animal laboratories.

## Conclusions and future directions

In conclusion, the current body of the literature suggests that sleep impairment generally increases autophagy, although tissue-specific differences are likely to be at play. Conversely, autophagy disruption can either increase or decrease total sleep time, possibly depending on autophagosome levels. Despite these insights, significant methodological limitations remain, including inadequate measurement of autophagy levels and flux, and suboptimal models for inducing sleep disturbances. Consequently, many questions about the relationship between sleep and autophagy remain open. Does autophagy play a different role during sleep and wake? What local mechanisms underlie tissue-specific differences in sleep-related autophagy? How is autophagy affected by sleep disturbances, and how is sleep affected when autophagy is impaired? Are other forms of selective autophagy beyond mitophagy regulated by sleep? Is the increase in autophagy after sleep impairment harmful, “a bystander” or a healthy response to a stressor? Can therapeutic strategies that modulate autophagy mitigate some of the adverse effects of sleep deprivation? Can sleep therapies improve autophagy flux? To properly answer these questions, future research requires robust experimental design. This includes choosing sleep impairment models that minimize confounding variables. Emerging techniques such as chemogenetics, optogenetics, and gene editing hold promises for advancing the field. Furthermore, phylogenic and development studies may offer additional opportunities. Accurate assessment of autophagy requires comprehensive measurements that capture flux - and preferably cargo sequestration or degradation - through multiple methods. Answers to these questions are more and more pressing given the rise of sleep disturbances in our modern societies. Understanding the autophagic function of sleep may be a very promising target to open new avenues of research for treatment of comorbid pathologies of sleep disturbance.

## Figures and Tables

**Table 1:** Autophagy methods.

Method	Strengths	Drawbacks	Ref
*Transmission electron microscopy* (TEM)	- Detects autophagic structures directly with great resolution, in their natural environment in relation to other organelles, allows for the quantification of these structures. - Immuno-EM can help with identification, although this is dependent on available antibodies and may introduce other artifacts.	- Effect of the slicing on the visualization of the structures. - Organelle identification requires expertise. - Low throughput.	40, 213
*Immunohistochemistry* (IHC) *Immunofluorescent imaging* (IF)	- Widely used to observe autophagic vesicles as LC3 positive puncta. - Levels and distribution of many other autophagy proteins can also be assessed. - Should be used in combination with lysosomal blockade to measure flux.	- Provides only snapshots of the autophagy state. - Necessitates target-specific antibodies. - LC3 puncta may reflect non-canonical functions.	40
*Western blotting (immunoblotting)*	- Widely used to measure lipidation of the ATG8 protein family members, such as LC3, since the lipidated form (referred to as LC3-II, see Fig. 1) migrates faster than the unlipidated form (LC3-I). - Levels and post-translational modifications of many other autophagy proteins can also be assessed. - Should be used in combination with lysosomal blockade to measure flux.	- Provides only snapshots of the autophagy state. - Necessitates target-specific antibodies. - ATG8 lipidation may reflect non-canonical functions.	40, 217
*Fluorescence-based flux assays* - LC3 or autophagic cargo fused with a tandem fluorescent probe (such as mCherry-GFP); or with the pH-sensitive protein Keima	- Fluorescence from LC3 associated with phagophores and autophagosomes distinguishable from fluorescence under the low pH in the lysosome. - Time-resolved measurements of dynamic changes possible.	- Requires transgenic animals (already exist in several species), transduction or similar. - Quantification and interpretation not always straight-forward.	40, 214, 215, 217
*LDH sequestration and long-lived* *protein degradation assays*	- Quantitative measurement of actual cargo sequestration or degradation.	- Cells are lysed or disrupted and therefore the exact same cells or tissues cannot be monitored over time.	216
*Quantitative polymerase chain* *reaction* (qPCR)	- Quantitative measurement of gene expression.	- Changes in transcription levels may not directly correlate with changes in autophagy and is an insufficient measure to infer actual changes in autophagic activity.	40

**Table 2:** Sleep manipulation methods

Category	Method	Description / Example	Comments	Literature example
Behavioral (passive)	Environmental or cognitive stress	Sleep disruption via increased stress or cognitive demand	Non-specific; stress may confound results	89
Mechanical (active)	Water tank / platform method	Animal placed on small platform above water; falls into water upon sleep	Originally REM-specific, but stressful and lacks precise control	146, 208, 209
	Modified Multiple Platform Method (MMPM)	Multiple animals in cage with several platforms	Most commonly used in rodent autophagy studies; more social and less restrictive	210
	Gentle handling	Sleep deprivation by light tactile stimulation	Less stressful but time-consuming and hard to standardize	207
	Automated poking/shaking devices	Devices stimulate the animal or shake the cage at set times or in response to activity	Allows closed-loop and circadian-specific manipulation; still may disturb controls	141,158
	Forced locomotion (e.g. treadmill, rotating wheels, sweeper arm)	Animal kept awake through constant movement	Impacts metabolism and stress response	53, 154, 159, 165, 166, 174, 211
	Sensory stimulation (e.g. light, temperature, odors)	Used to modulate sleep indirectly	Increased temperature affects day/night sleep in flies, less is known about autophagy impact	212
Pharmacological	Modafinil	Promotes wakefulness	Used in clinical and occupational settings	153
	Propofol or H(2)S	Induces sleep/sedation	Sleep-promoting agents used experimentally	151, 152, 171
Lesions / physical interventions	Tympanic membrane surgery	Induces vestibular damage affecting sleep	Invasive; limited use in autophagy-related studies	182
Genetic models	Sleep apnea models via gene manipulation	Targeted gene models for sleep disorders	Useful for disease modeling	194
	Long/short-sleeping mutants	Mutants identified by sleep phenotype	Not always linked to sleep-regulatory genes	141, 143

## Box

Autophagy and sleep play contrasted and complementary roles in maintaining homeostasis.

Sleep impairment increases autophagy, but whether this is beneficial or detrimental is still debated.

Levels of autophagosome may modulate sleep amount
